# Assessing and managing the risks of COVID-19 in the workplace: Applying industrial hygiene (IH)/occupational and environmental health and safety (OEHS) frameworks

**DOI:** 10.1177/0748233720967522

**Published:** 2020-10-21

**Authors:** Rachel E Zisook, Andrew Monnot, Justine Parker, Shannon Gaffney, Scott Dotson, Kenneth Unice

**Affiliations:** 1 205740 Cardno ChemRisk, San Francisco, CA, USA; 2 205740 Cardno ChemRisk, Boulder, CO, USA; 3 205740 Cardno ChemRisk, Cincinnati, OH, USA; 4 205740 Cardno ChemRisk, Pittsburgh, PA, USA

**Keywords:** IH/OEHS frameworks, COVID-19, SARS-CoV-2, risk assessment, risk management

## Abstract

As businesses attempt to reopen to varying degrees amid the current coronavirus disease (COVID-19) pandemic, industrial hygiene (IH) and occupational and environmental health and safety (OEHS) professionals have been challenged with assessing and managing the risks of COVID-19 in the workplace. In general, the available IH/OEHS tools were designed to control hazards originating in the workplace; however, attempts to tailor them specifically to the control of infectious disease outbreaks have been limited. This analysis evaluated the IH decision-making framework (Anticipate, Recognize, Evaluate, Control, and Confirm (“ARECC”)) as it relates to biological hazards, in general, and to severe acute respiratory syndrome coronavirus 2 (SARS-CoV-2), specifically. Available IH/OEHS risk assessment and risk management tools (e.g. control banding and the hierarchy of controls) are important components of the ARECC framework. These conceptual models, however, were primarily developed for controlling chemical hazards and must be adapted to the unique characteristics of highly infectious and virulent pathogens, such as SARS-CoV-2. This assessment provides an overview of the key considerations for developing occupational infection control plans, selecting the best available controls, and applying other emerging tools (e.g. quantitative microbial risk assessment), with the ultimate goal of facilitating risk management decisions during the current global pandemic.

## Introduction

Severe acute respiratory syndrome coronavirus 2 (SARS-CoV-2) emerged in Wuhan province, China, in late 2019 and is the virus that causes coronavirus disease of 2019 (“COVID-19”) (WHO, 2020a). The World Health Organization declared COVID-19 a pandemic in March 2020 (WHO, 2020b). Available data indicate that the majority of individuals infected with SARS-CoV-2 experience mild or moderate respiratory illness and are able to recover without treatment (CDC, 2020a). However, certain subsets of the population, such as elderly adults and those with underlying health conditions (e.g. chronic respiratory disease, cardiovascular disease, and diabetes), appear to be at a greater risk of developing serious illness, which may lead to death (CDC, 2020c).While SARS-CoV-2 is well recognized for causing pulmonary disease, infection can also result in neurological, renal, hepatic, gastrointestinal, cardiovascular, endocrine, thromboembolic, and dermatological extrapulmonary effects (Gupta et al., 2020).

As of October 14, 2020, a total of 38,423,591 COVID-19 cases and 1,091,123 deaths have been reported globally (JHU, 2020). In the United States, total confirmed and probable cases have reached 7,909,035, and 216,639 deaths have been reported (JHU, 2020). Beyond the significant global burden of COVID-19, the current pandemic has resulted in unprecedented economic hardship. The recommended practices of social/physical distancing, self-isolation, and other measures implemented as responses across the United States have resulted in a decreased workforce in nearly all sectors of our economy and the loss of countless jobs. Although SARS-CoV-2 continues to spread throughout the population, states are attempting to reopen to varying degrees. As the knowledge and understanding of SARS-CoV-2 and COVID-19 continue to evolve, governments and employers need to begin preparing for employees to return to the physical workplace. Over the years, teams of interdisciplinary scientific experts have come together to develop tools for employers and governments to help mitigate virus transmission, particularly in the workplace.

Industrial hygienists and other occupational and environmental health and safety (OEHS) professionals are skilled in controlling biological, chemical, and physical hazards in occupational settings. These professionals often receive academic training in multiple disciplines and are equipped with established tools and frameworks for assessing and controlling workplace hazards. The American Industrial Hygiene Association (AIHA) states that the role of an OEHS professional within an organization is to offer guidance on the “Anticipation, Recognition, Evaluation, Control, and Confirmation of industrial hygiene and environmental stressors in, or arising from, the workplace that may result in injury, illness, impairment, or affect the well-being of workers and members of the community” (AIHA, 2020). More specifically, the role of the industrial hygienist in a pandemic “is to provide advice and recommendations on control measures for the workplace and community (i.e., administrative controls, personal protective equipment (PPE), and engineering), in coordination with the infection prevention and control specialist, based on the best available information” (AIHA, 2006: 1). This description holds true for professionals challenged with assessing and managing COVID-19 risks in the workplace and, by extension, the global community currently impacted.

General frameworks and tools are available to professionals for assessing and managing the risks of various hazards in the workplace, such as the Anticipate, Recognize, Evaluate, Control, and Confirm (ARECC) decision-making framework, risk assessment, the National Institute for Occupational Safety and Health (NIOSH) hierarchy of controls, and control banding. While these concepts and tools have been applied successfully to biological hazards in the workplace, attempts to tailor them specifically to controlling infectious disease outbreaks have been limited. Further, there are additional concepts that need to be considered, such as the Chain of Infection, which may help to mitigate viral transmission in the workplace. While general resources and guidance exist for industrial hygiene (IH) and OEHS professionals during a pandemic, the current COVID-19 pandemic warrants unique considerations. As such, the purpose of this article is to provide an overview of the tailored application of available IH and OEHS risk assessment frameworks, tools, and management principles to COVID-19.

## Applying IH/OEHS frameworks to COVID-19

### The IH decision-making framework

**Figure 1.**
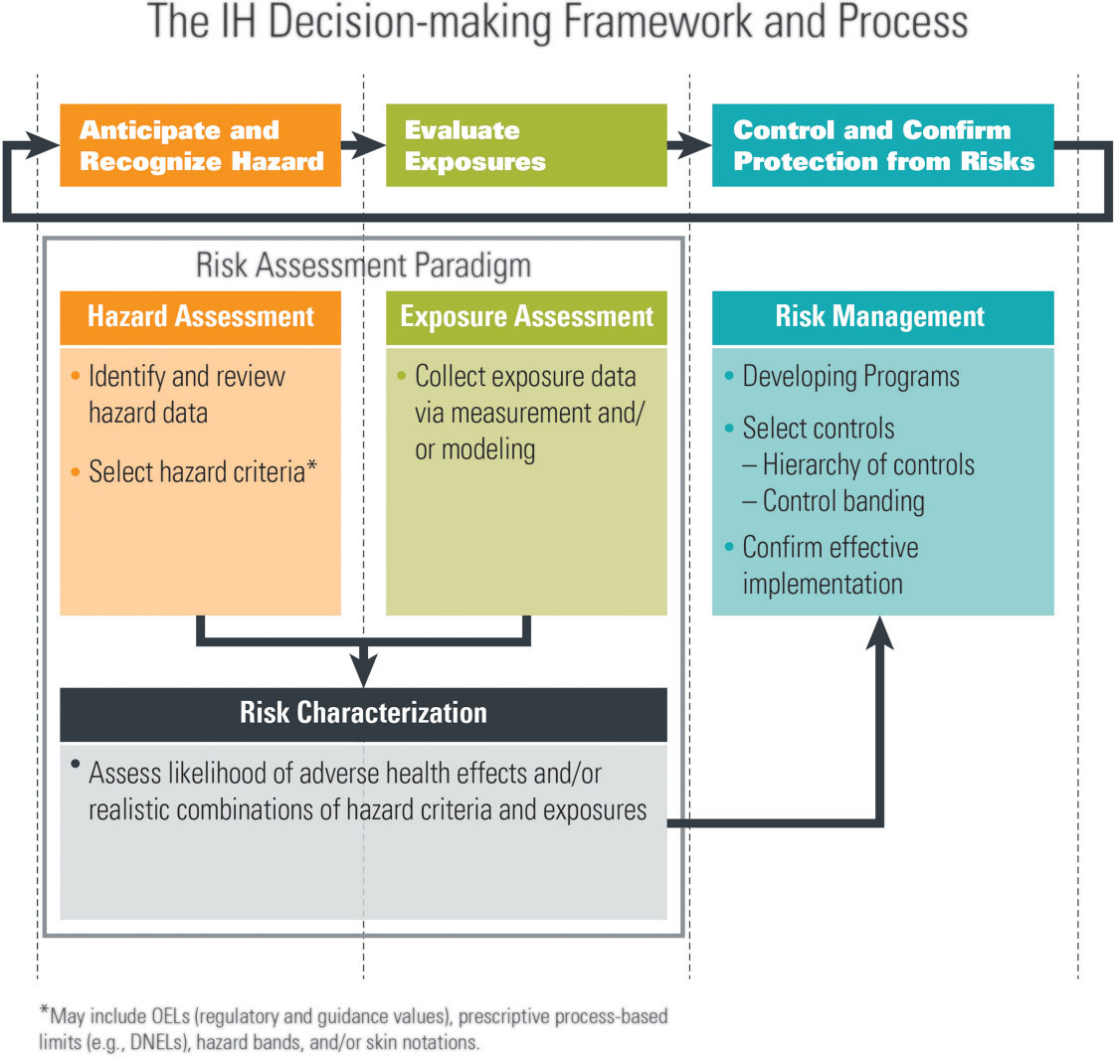
The IH decision-making framework and process. *Source*: Adapted from Jahn et al. (2015). IH: industrial hygiene.

The IH decision-making framework is used to anticipate and recognize chemical, physical, and biological hazards in the workplace; evaluate potential exposures; and ultimately, control and confirm that workers are protected from risks (“ARECC”) (Figure 1). This tool can be utilized to inform and drive corporate risk management decisions regarding potential occupational hazards. The major steps of this framework (Risk Assessment and Risk Management) and its key components (see Figure 1) are described below as they relate to biological hazards, in general, and to SARS-CoV-2, specifically.

### Risk assessment

The AIHA model for risk assessment is consistent with standard risk assessment protocols for chemical hazards established by the National Research Council (Jahn et al., 2015; NRC, 2009). Compared with chemical risk assessment, occupational risk assessment for biological hazards is particularly challenging because of the high level of variability in exposures, sampling method limitations, differences in worker susceptibility, and a lack of epidemiological data to support developing occupational exposure limits (Carducci et al., 2016). As such, while chemical and physical agents are frequently evaluated on a quantitative basis, a qualitative or semi-quantitative approach is typically employed for biological agents (AIHA, 2006; Carducci et al., 2016).

Currently, there are four qualitative risk groups (RGs) for biological agents determined as a function of the pathogenicity (Table 1). These were established by the National Institutes of Health (NIH) and adopted by AIHA in 2006 (AIHA, 2006; NIH, 2019b). Researchers have previously utilized RGs as surrogates for toxicity when characterizing the risk levels of workers exposed to aerosol-transmissible infectious diseases (Sietsema et al., 2019). These RGs consider qualitative variations in factors both inherent to the current status of the disease, as well as temporal changes caused by mutations in prevalent viral stains, and modifications in pharmaceutical interventions or disease prevalence in the community.

**Table 1.** Qualitative RGs for biological agents.

Qualitative group	Individual risk	Community risk	Definition
RG1	Low	Low	Agents that are not associated with disease in healthy adult humans
RG2	Moderate	Low	Agents that are associated with human disease which is rarely serious; preventive or therapeutic interventions are often available
RG3	High	Low	Agents that are associated with serious or lethal human disease for which preventive or therapeutic interventions may be available (high individual risk but low community risk)
RG4	High	High	Agents that are likely to cause serious or lethal human disease for which preventive or therapeutic interventions are not usually available (high individual risk and high community risk)

Note. Adapted from AIHA (2006) and NIH (2019b).

NIH: National Institutes of Health; AIHA: American Industrial Hygiene Association; RG: risk group.

### Hazard assessment: SARS-CoV-2

As reported by the NIH, the current Interim Laboratory Biosafety Guidance states that SARS-CoV-2 and other coronavirus strains known to cause severe infections (SARS-CoV and MERS-CoV) are classified as RG3 viruses (NIH, 2019a, 2019b). Differences in assigning risk categories for laboratories as compared to other workplace settings may exist, however. Furthermore, the RG may change over time as the level of individual and community risk evolves in response to fluctuations in the prevalence of community spread, the potency of the circulating strains, and/or pharmaceutical interventions.

As noted above, “community” and “individual” risks are key components of the NIH framework, each of which can be qualitatively rated at one of three levels (low, moderate, and high). Indicators of community and individual risks may need to be adapted, however, when conducting occupational risk assessments for SARS-CoV-2. For example, NIH RG3 applies to agents for which preventive or therapeutic interventions “may be available” (NIH, 2019a: 42). While such interventions may be available for SARS-CoV-2 in some parts of the globe, they may or may not be on a more local scale. As such, in using the NIH risk groupings for occupational risk assessment purposes, it may be prudent to consider which risk grouping for SARS-CoV-2 is most appropriate at the national, state, regional, and city levels. Fortunately, several community risk assessment tools have emerged with key risk indicators, including tools developed to estimate the likelihood of contacting COVID-positive individuals based on event size (see Resources). In terms of “individual” risk, the NIH framework may also need to be adapted to account for individual susceptibility. For example, the RG3 category specifies that individuals have “high” individual risk; however, recent experience has shown that some individuals infected with SARS-CoV-2 are asymptomatic or experience mild symptoms, while others may be at increased risk of more serious health outcomes (WHO, 2020c).

### Exposure assessment: SARS-CoV-2

At present, there are considerable uncertainties in characterizing exposure to bioaerosols, including SARS-CoV-2, largely owing to methodological sampling limitations. As such, assessing occupational biological exposure is typically limited to a qualitative characterization (e.g. low, medium, and high) (Carducci et al., 2016; Sietsema et al., 2019). Key variables for characterizing exposure are dependent upon the mode of transmission. Current scientific consensus is that COVID-19 is transmitted primarily through exposure to respiratory droplets (>5 µm in diameter) via the mouth, nose, or eyes from direct contact with an infected individual (CDC, 2020a; WHO, 2020c). Evidence from laboratory and epidemiology studies suggests that airborne transmission may also contribute to COVID-19 spread under certain conditions. This mode of transmission refers to droplets less than 5 µm in diameter, commonly referred to as “droplet nuclei,” “aerosols,” or “microdroplets,” that may remain suspended in air for extended periods and pose a risk of exposure beyond the immediate vicinity of an infected individual (Bourouiba, 2020; Howard et al., 2020; Morawska and Milton, 2020; WHO, 2020c; Zhang et al., 2020). Transmission of SARS-CoV-2 may also occur indirectly through fomite transmission, in which individuals are exposed to the virus from touching surfaces contaminated by settled respiratory secretions or droplets from infected individuals, followed by touching the mouth, nose, or eyes (WHO, 2020c). Other modes of transmission are possible, including fecal–oral, blood-borne, and animal-to-human; however, data on these are currently limited (WHO, 2020c).

Sietsema et al. (2019) proposed to qualitatively characterize exposure (*E*) to aerosol-transmissible infectious diseases based on the likelihood (*L*) and duration (*D*) of exposure to potentially infectious individuals (*E* = *L* × *D*). This general equation can be applied to SARS-CoV-2 to characterize exposure via (a) direct contact and droplets and (b) airborne transmission. The variables that drive exposure differ for these modes, however. For example, in scenario “a,” exposure is characterized as being within a short range (e.g. less than 6 feet) of an infectious individual. In scenario “b,” exposure may occur both at short range and at greater distances. Key factors that may influence the likelihood and duration of exposure to smaller respiratory droplets responsible for airborne transmission include number and location(s) of potentially infectious individuals and environmental losses through ventilation and other means. Environmental losses may be dictated by room size, air changes per hour, air distribution patterns, room pressurization, temperature, humidity, percent outdoor air, and air filtration/treatment (e.g. ultraviolet germicidal irradiation). The equation proposed by Sietsema et al. (2019) is not applicable to fomite transmission. Exposure to SARS-CoV-2 from surfaces may be assessed based on human behaviors (e.g. hand hygiene and face touching), however, and the likelihood for contact with contaminated surfaces (a function of the frequency with which surfaces are touched, cleaning and disinfection efficacy, residence time of SARS-CoV-2 on surfaces, and glove-wearing practices).

### Risk management

After characterizing the hazard and potential risk that a pathogen poses to workers, strategies must be implemented to mitigate workplace transmission. Standards and regulations for managing the risks of infectious disease transmission have previously been established for occupational settings. For example, the Joint Commission on Accreditation of Healthcare Organizations (JCAHO), an independent, nonprofit organization that evaluates and accredits health-care organizations and programs in the United States, has developed standards designed to aid health-care organizations in the prevention and control of infection (JCR, 2015). In addition, the Occupational Safety and Health Administration (OSHA) has developed standards and directives applicable to protecting workers from infectious diseases, including the Bloodborne Pathogens (BBP) standard (29 CFR 1910.1030), issued in 1991 (OSHA, 2000). Although the BBP standard has been effective in protecting workers from blood-borne pathogens, it does not address infectious diseases transmitted by other routes (e.g. contact, droplet, and airborne). In recognition of this gap, OSHA is currently developing a standard to ensure that employers establish a comprehensive infection control program and control measures to protect employees from infectious disease exposures to pathogens capable of causing significant disease (OSHA, 2017, 2020b).

The available infection control standards developed by JCAHO and OSHA can be applied to develop COVID-19 mitigation measures. For example, although specific to the health-care industry, JCAHO provides relevant guidance for planning, implementing, and improving infection control plans (JCR, 2015). However, given that the available standards do not specifically address risk management for pandemics, it is necessary to consult supplemental guidance for COVID-19. In its 2006 guidelines, AIHA emphasized the importance of developing plans to address pandemic preparedness and response, business continuity, and communications (AIHA, 2006). In 2009, OSHA similarly stressed the need to develop plans for pandemic preparedness in its Guidance on Preparing Workplaces for an Influenza Pandemic. The agency cited the General Duty Clause, requiring “employers to provide their employees with a workplace free from recognized hazards likely to cause death or serious physical harm” (OSHA, 2009: 3). OSHA also called for employers to identify risk levels in workplace settings and select appropriate control measures (OSHA, 2009).

**Figure 2.**
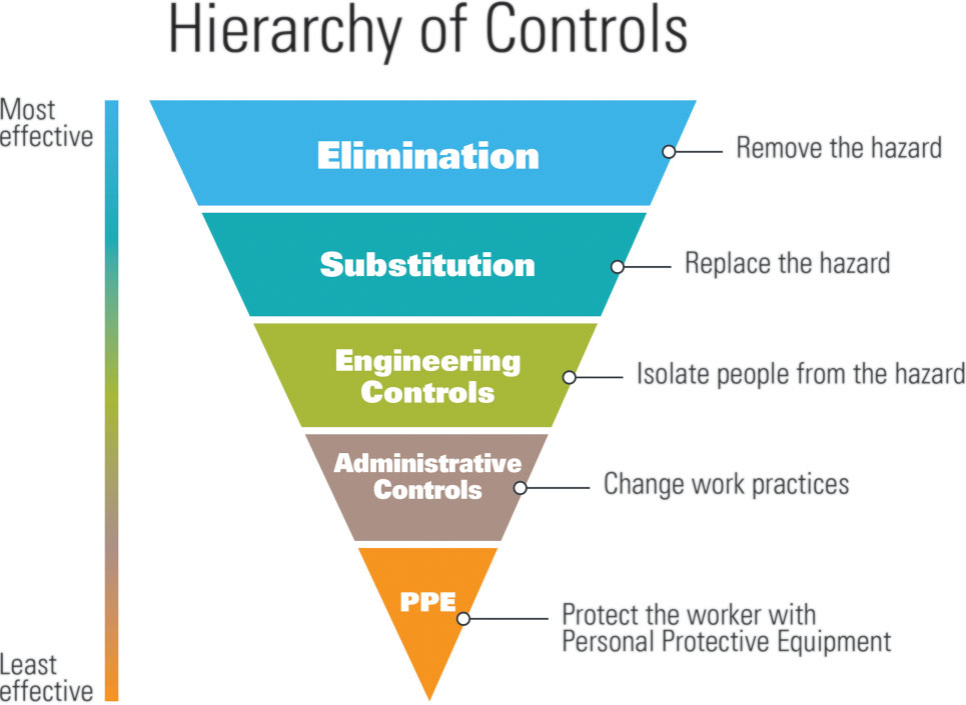
The hierarchy of controls. *Source*: Adapted from NIOSH (2015).

**Figure 3.**
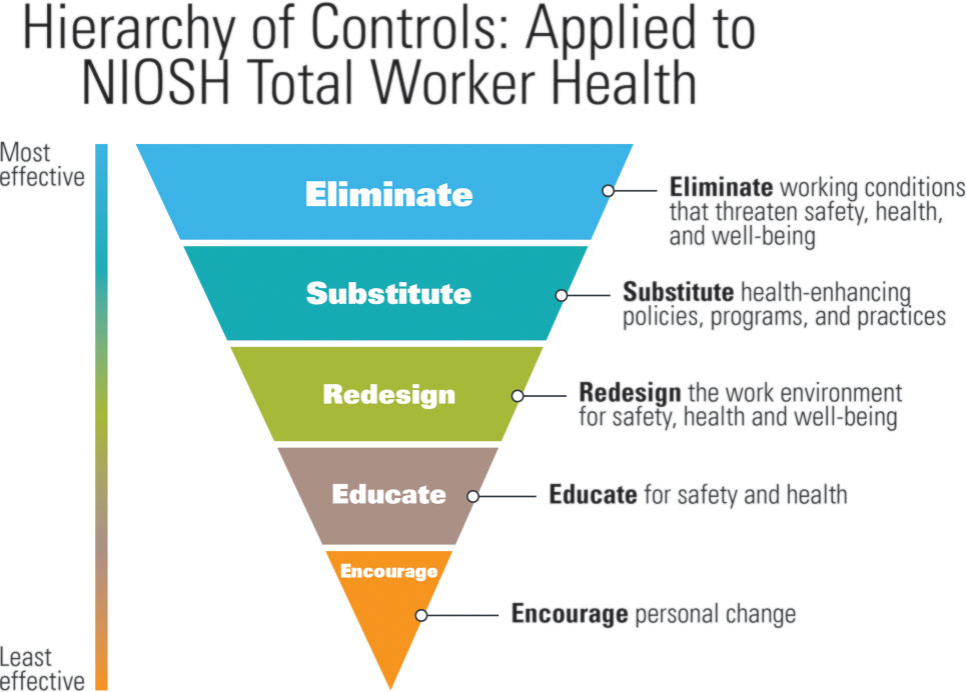
The hierarchy of controls applied to TWH. *Source*: Adapted from NIOSH (2018). TWH: Total Worker Health.

OSHA and the US Centers for Disease Control have provided rolling strategies and recommendations for employers in light of the current COVID-19 pandemic (CDC, 2020b; OSHA, 2020a). Broadly, these consist of various engineering controls, administrative processes, and employee use of appropriate PPE (CDC, 2020b; OSHA, 2020a). These strategies are generally consistent with the current NIOSH hierarchy of controls utilized to mitigate occupational exposure to hazards (Figure 2) and the NIOSH hierarchy of controls applied to Total Worker Health (TWH) (Figure 3). In the absence of effective evidence-based guidelines, employers can consider implementing control banding for SARS-CoV-2. This approach draws on the hierarchy of controls but adds another dimension to the fold: assigning jobs and/or job tasks into specific risk categories, and then subsequently prioritizing the application of controls. In 2019, researchers proposed such a strategy to control a viral pathogen transmitted via aerosols in the workplace (Sietsema et al., 2019).

Control banding and the NIOSH hierarchy of controls are useful tools for developing risk management plans, including selecting controls. However, these conceptual models were primarily developed for controlling chemical hazards and, in many respects, are ill-equipped to address highly infectious and virulent pathogens, such as SARS-CoV-2. Although not specific to physical, chemical, or biological hazards, the NIOSH hierarchy of controls adapted to TWH is a helpful supplement to the available IH/OEHS frameworks. The NIOSH TWH approach is defined as “policies, programs, and practices that integrate protection from work-related safety and health hazards with promotion of injury and illness–prevention efforts to advance worker well-being” (NIOSH, 2016: 1). The TWH approach acknowledges that risk factors in the workplace can contribute to health problems previously considered unrelated to work and stresses the need for integrated interventions (NIOSH, 2016). These concepts are fundamental to managing the risks of COVID-19, which cross work/life boundaries, and necessitate a multilayered approach to preventing illness and advancing worker well-being. The following sections outline the key components of risk management plans based on the available frameworks and standards and methods for adapting existing IH/OEHS tools to inform control selection for COVID-19.

### COVID-19 risk management plans

Examples of risk management plans for COVID-19 include Infection Control plans and Return to Work plans. Most businesses, with the exception of those subject to the standard requirements of JCAHO or the OSHA BBP regulation, likely have not had infection control on their top list of risks to consider when creating risk management plans. Such controls, however, will no doubt be incorporated into company risk management and business continuity planning for the foreseeable future. Infection Control Plans for COVID-19 can vary across businesses; however, at a minimum, they should address Roles and Responsibilities, Communication, Worker Behavior and Hygiene, Facility Cleaning and Disinfection, Reporting, Product Handling, and Case Management. Definitions of these topics will vary by industry, and the degree to which customer contact occurs will alter the layers of controls needed.

In comparison, Return to Work plans typically include a decision matrix that relies on the foundational measures set forth in the Infection Control Plan. A typical Return to Work plan creates a phased or staged approach, with varying levels of controls for each phase/stage. Such plans typically consist of three stages: “Full Infection Control Response” (Stage 1), “Modified Infection Control Response” (Stage 2), and “Normal Business Operations” (Stage 3). Along with these defined stages and their scaled controls, defined triggers should be put in place to clearly specify when a facility or company can transition from one stage to another. Typically, trigger evaluation is based on epidemiological metrics and can include local case counts, hospitalizations, deaths, rate of change over 7 days, case counts within a facility, and local or state recommendations and their relative stages.

When designing and implementing such plans, secondary hazards and risks may be introduced and should be anticipated and evaluated. Additional hazards may include respiratory and skin hazards associated with antimicrobial agents, heat stress associated with improper PPE use or lack of training, physical stress or fatigue from heightened responses when working during a pandemic, or mental health impacts from ongoing concerns regarding potential exposures and health risks to self and family.

### Selection of controls to reduce the risk of COVID-19 transmission

As noted above, OSHA recommended that employers identify risk levels in workplace settings and select appropriate control measures (OSHA, 2009). Control banding is a useful IH/OEHS framework for establishing risk levels. Although typically employed for chemical hazards that lack occupational exposure limits, risk levels for SARS-CoV-2 can be determined via a combination of the hazard (e.g. NIH RG) and the exposure rating (e.g. high, medium, and low). Appropriate controls can then be selected for a given risk level.

The IH/OEHS hierarchy of controls has been widely applied to reduce workplace risk associated with new agents derived from previously studied materials such as nanomaterials (Schulte et al., 2008). COVID-19 transmission, however, is meditated by a novel pathogen lacking options for some of the preferred control methods such as agent substitution or essential worker contact elimination. Recently, in consideration of the challenges associated with novel pathogens, Sietsema et al. (2019) recommended a complementary risk management framework consisting of (1) source, (2) pathway, and (3) receptor. Workers represent potential (mobile) sources of SARS-CoV-2-containing respiratory droplet “emissions,” and, simultaneously, controls must be implemented to mitigate transmission pathways between employees. An example that illustrates the key considerations and data gaps associated with each of these three types of controls is presented in Table 2.

**Table 2.** Example illustrating the evaluation of key considerations and data gaps for the three key source control option categories for a novel respiratory pathogen.

Control type	Description	Control option	Key considerations	Data gaps
Source	Remove or reduce exposure at the source	Social/physical distancing	“6 ft. rule” is not a hard line; respiratory droplets may travel greater distances, particularly indoors	Fate and transport of bioaerosols containing viable SARS-CoV-2
		Screening (e.g. wellness questionnaires, and temperature checks)	Some individuals may be presymptomatic/asymptomatic	
		Testing	Accuracy depends on test type; no test is 100% accurate	
		Case investigation and contact tracing	Must define what constitutes a “close contact”; protect case confidentiality; ensure collaboration and communication with public health departments	
		Face coverings	Fit; fabric type; laundering/replacement rate; decrease in effectiveness with use; worker behaviors; comfort; perception (e.g. false sense of security)	Effectiveness of reducing respiratory droplet emissions under various conditions and for various fabric types; effectiveness at reducing transmission
		Product isolation	Different considerations for different surfaces	Residence time on various surfaces Likelihood of fomite transmission
Pathway	Interrupt pathway between source and infected individuals	HVAC	Key factors that could influence airborne transmission include air flow; pressurization; filtration; treatment (e.g. UVGI); occupancy (number and location of people)	Relative effectiveness of various options
		Cleaning and disinfection	Surfaces must first be cleaned, and then disinfected (sanitizing products are insufficient for SARS-CoV-2); EPA list N; potential for exposure to antimicrobial agents; perception (e.g. false sense of security); relative contribution of fomite transmission	Effectiveness
		Barriers (e.g. plexiglass)	Location and height	Effectiveness
		Hands-free high touch surfaces (e.g. hand dryers)	Potential interference with general ventilation	Extent to which hand dryers may interfere with general ventilation
		Worker behavior and hygiene	Accessibility to hand washing/sanitizing stations; compliance; training	
Receptor	Minimize exposure at the receptor (worker).	PPE	Shortages; effectiveness; fit testing and RPP for respirators; reuse; decontamination/disposal; training/education (e.g. donning/doffing); risks of wearing	Effectiveness

HVAC: heating, ventilation and air-conditioning; EPA: environmental protection agency; PPE: personal protective equipment; RPP: respiratory protection program; UVGI: ultraviolet germicidal irradiation.

**Figure 4.**
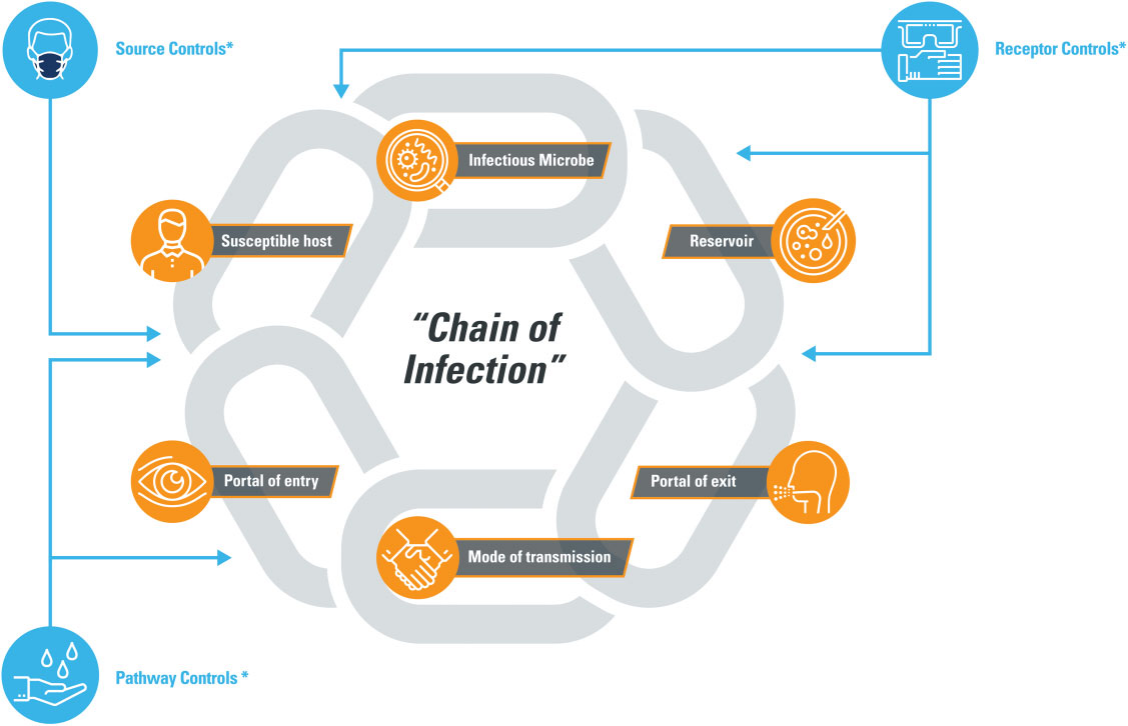
Breaking the “Chain of Infection.” *Controls outlined in Table 2.

The “hierarchy” concepts for selecting controls must be applied with caution, however, because multiple types of controls will be necessary to mitigate workplace disease transmission in instances in which eliminating the source from the workplace (i.e. workers stay home) or isolating workers at great distances (e.g. separate buildings) is not feasible. The “Chain of Infection” is a cornerstone of infection prevention and is a useful concept for conceptualizing interacting factors that contribute to pathogen transmission such as SARS-CoV-2. As illustrated in Figure 4, multiple controls, including source, pathway, and/or receptor controls, should be employed at strategic locations intended to “break the chain” of transmission. Control selection must also consider comfort and accessibility and should accompany a clear understanding and ability to communicate the reasons for selection in order to ensure successful implementation.

While the available risk management tools are simple in concept, in practice, decision-making requires careful consideration of uncertainty and variability, such as the lack of data on the effectiveness of various controls, limited availability or feasibility of some controls, and differences in perception of risk among individuals and communities. Additionally, as described above, challenges are inherent when assessing “hazard” and “exposure” for SARS-CoV-2, resulting in qualitative and low resolution level in characterizing risk (e.g. low, medium, and high).

Researchers are actively working toward filling data gaps with continued laboratory and epidemiologic research, as evidenced by the 168,489 research publications and 6,199 clinical trials related to the study of SARS-CoV-2 or COVID-19 in 2020 as of October 14, 2020 (Dimensions, 2020). One notable area of investigation with potential relevance to airborne pathogens is the application of quantitative microbial risk assessment (QMRA) methodologies to inform risk management tools. For example, QMRA methods have been applied to identify specific occupational settings and exposure profiles with the highest likelihood of adenovirus transmission (Carducci et al., 2016) and MERS-CoV (Adhikari et al., 2019). Specific to SARS-CoV-2, QMRA methods have been used to characterize the relative contributions of transmission routes among health-care personnel (Jones, 2020) and the potential role of wastewater in SARS-CoV-2 transmission (Kitajima et al., 2020). The available dose-response and exposure data for SARS-CoV-2 are limited, necessitating using assumptions and surrogate data when applying QMRA methodologies. These approaches, however, can still be used as tools for understanding the potential drivers of exposure and risk.

## Summary and conclusions

Established infectious disease standards and IH/OEHS frameworks can be adapted to address the unique considerations of SARS-CoV-2 and the modern workplace. Reliance on these frameworks should consider that the “traditional” workplace paradigm has shifted to one in which community considerations play a significant role. Currently, aside from health-care settings, there are no established occupational health standards for assessing and managing the risks of exposure to pathogens responsible for active community outbreaks in the United States. While the health risk assessment paradigm is well established for chemical hazards in the workplace, significant challenges exist when applying it to biological hazards responsible for infectious disease outbreaks, such as COVID-19. As discussed here, there are limitations in assessing “hazard” and “exposure” for SARS-CoV-2, resulting in a qualitative and low level of resolution in risk characterization (e.g. low, medium, and high).

Despite these limitations, information obtained from the risk assessment process should serve as the basis for determining appropriate risk management actions for COVID-19, with the goal of mitigating viral transmission in the workplace, facilitating business continuity, and advancing worker well-being. IH/OEHS frameworks, adapted to the unique characteristics of SARS-CoV-2, are useful for developing evidence-based, risk management plans to accomplish these goals. Current challenges in selecting controls include lack of data on the effectiveness of various controls, limited availability of some controls, differences in individual and community perceptions of risk, and challenges regarding the feasibility of some interventions. To ensure successful control implementation, control selection must consider comfort and accessibility and should accompany a clear understanding and ability to communicate the reasons for selection. Over time, approaches for characterizing and managing SARS-CoV-2 risk will improve, as data continue to emerge and enhance our understanding of exposure and risk in occupational settings. In the future, best practices for assessing and managing the hazards of SARS-CoV-2 and other pathogens in the workplace should take an integrative approach, incorporating the best available scientific data in conjunction with psychosocial and community factors.

## Resources

International Laboratory for Air Quality and Health. Airborne Infection Risk Calculator. Available at: https://research.qut.edu.au/ilaqh/projects/expiratory-aerosols-and-infection-spread/ (accessed 14 October 2020).
University of Georgia COVID-19 Event Risk Assessment Tool. Available at: https://covid19risk.biosci.gatech.edu/ (accessed 14 October 2020).
WHO Mass Gathering COVID-19 Risk Assessment Tools. Available at: https://www.centerforhealthsecurity.org/our-work/publications/updated-who-covid-19-mass-gatherings-risk-assessment-tools (accessed 14 October 2020).
